# Supplementary material for: Regulatory perturbations of ribosome allocation in bacteria reshape the growth proteome with a trade-off in adaptation capacity
Source: iScience. 2022 Feb 7;25(3):103879. doi: 10.1016/j.isci.2022.103879 (PMC8866900; doi:10.1016/j.isci.2022.103879)
Supplement: Document S1. Figures S1–S8 [file mmc1.pdf]

iScience, Volume *25*

Supplemental information

Regulatory perturbations of ribosome allocation in bacteria reshape the growth proteome with a trade-off in adaptation capacity

David Hidalgo, César A. Martínez-Ortiz, Bernhard O. Palsson, José I. Jiménez, and José Utrilla

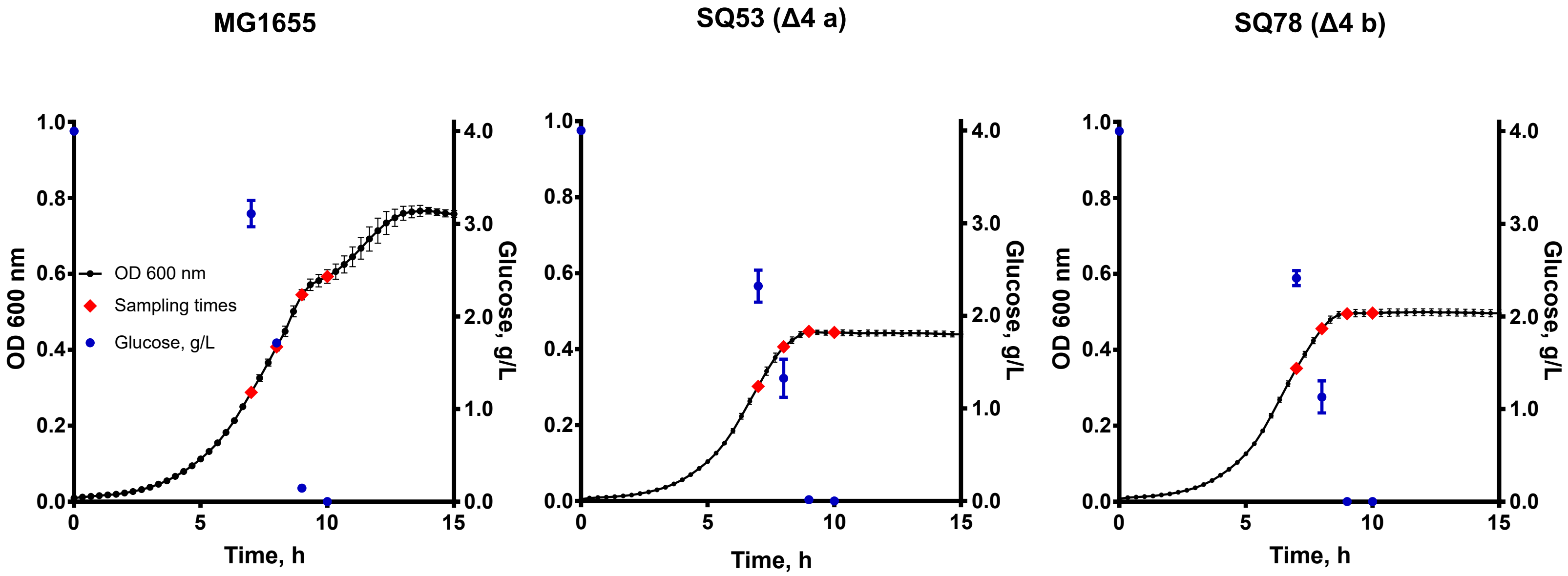

Figure S1. Related to figure 1D and 3. Glucose quantification. Glucose concentration was determined directly in supernatants of samples taken from MG1655, SQ53 and SQ78 during growth on glucose minimal medium. Samples were taken at t = 0h and close to the growth shift. Glucose is depleted at the growth shifts.

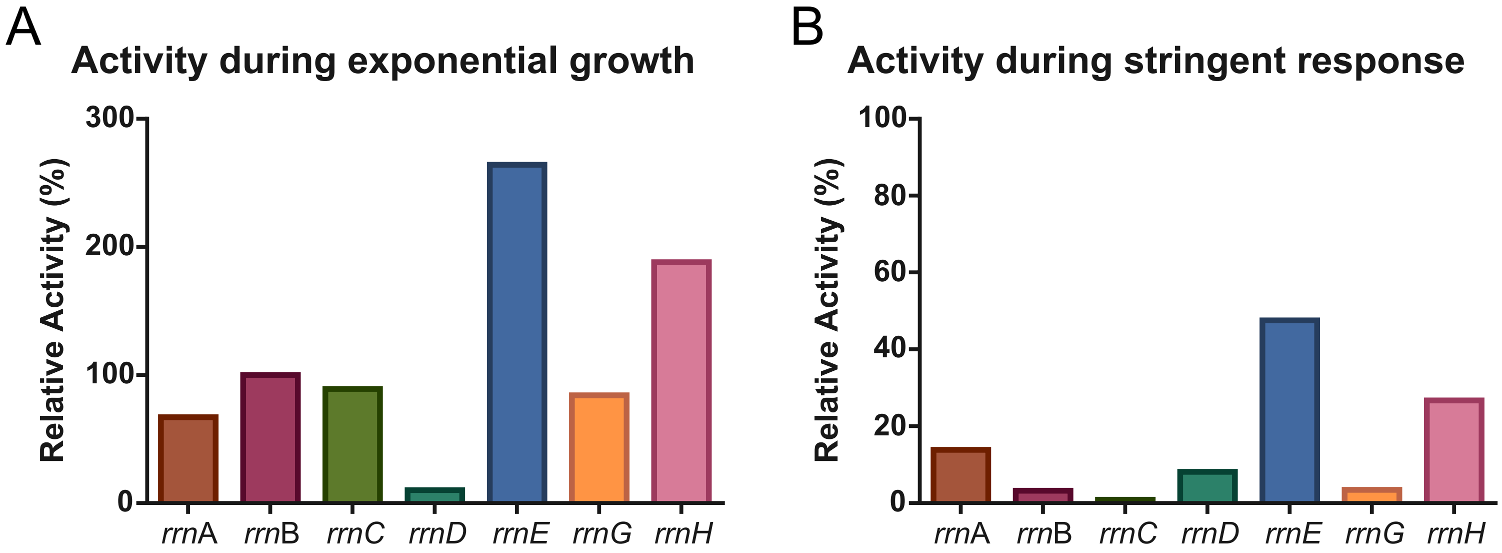

Figure S2. Related to Fig. 1, 2 and 3. Strength and sensitivity of P1 promoters towards ppGpp. Taken and adapted from Kolmsee et al., 2011 . Individual strengths of the rrn P1 promoters during A, exponential growth, and B, stringent response, as measured by a stable RNA product and relative to the *rrn*B P1 promoter. Panel A shows the heterogeneous activities among P1 promoters, ranging from 10% (*rrn*D) to 264 % (*rrn*E). Panel B shows reduced P1 promoter activities due to (p)ppGpp action, al so elucidating heterogeneous sensitivity to stringent response. SQ53, with 3 rrn copies and having the strongest but also the least sensitive P1 promoter (P1 *rrn*E) responds poorly to signals to reduce rRNA transcription (the major effect of stringent respon se). SQ78, with the same number of rrn copies but differing only in having *rrn*B instead of *rrn*E, has more responsive P1 *rrn* promoters to stop rRNA transcription. This allows the RNAP to be allocated in the transcription of other necessary genes for growth in a new carbon source and, hence, has a shorter time to resume exponential growth.

Normalized *rplI:msfGFP* fluorescence during exponential growth

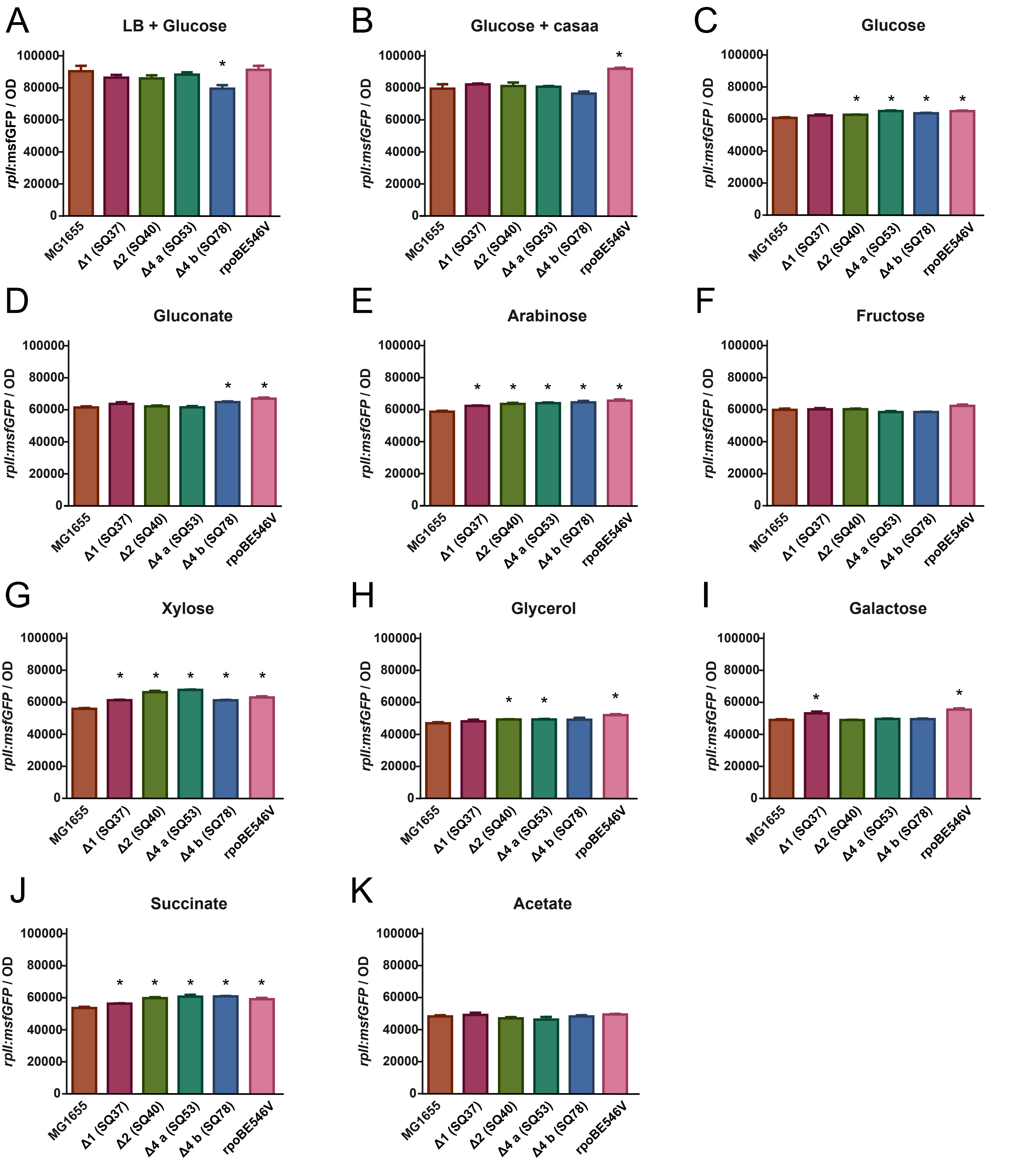

Figure S3. Related to Fig. 2. A-K, Normalized *rplI:msfGFP* fluorescence during exponential growth for mutants grown in different media. The normalized *rplI:msfGFP* fluorescence during early exponential growth was determined over several growth media for *rrn* and *rpoB* mutants. * denotes significant differences (95% confidence).

rplI:msfGFP and growth rate. Linear regressions

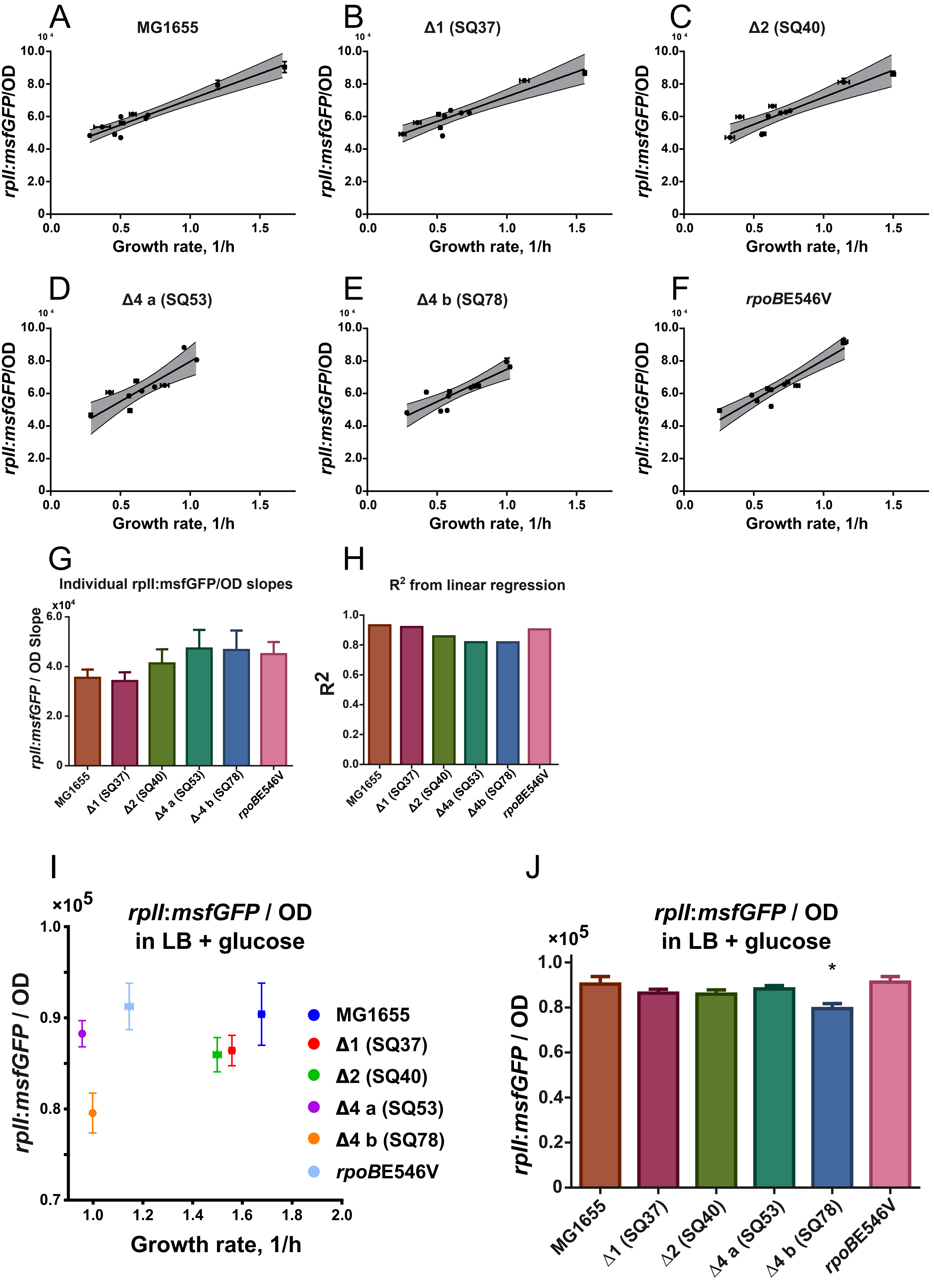

Figure S4. Related to Fig. 2. A-G, Linear regressions of normalized *rplI:msfGFP* as a function of growth rate for mutants grown in different media. Shadowed area represents the 95% confidence interval. H, Bar plot of slopes obtained through linear regression of the *rplI:msfGFP/OD* values for each growth medium for every strain. I, R2 values representing goodness of fit. I, Normalized fluorescence for strains grown in LB + glucose as a function of growth rate. J, Normalized fluorescence comparison between mutants and the WT in LB + glucose. Error bars represent standard deviations. * denotes significant difference with 95% confidence

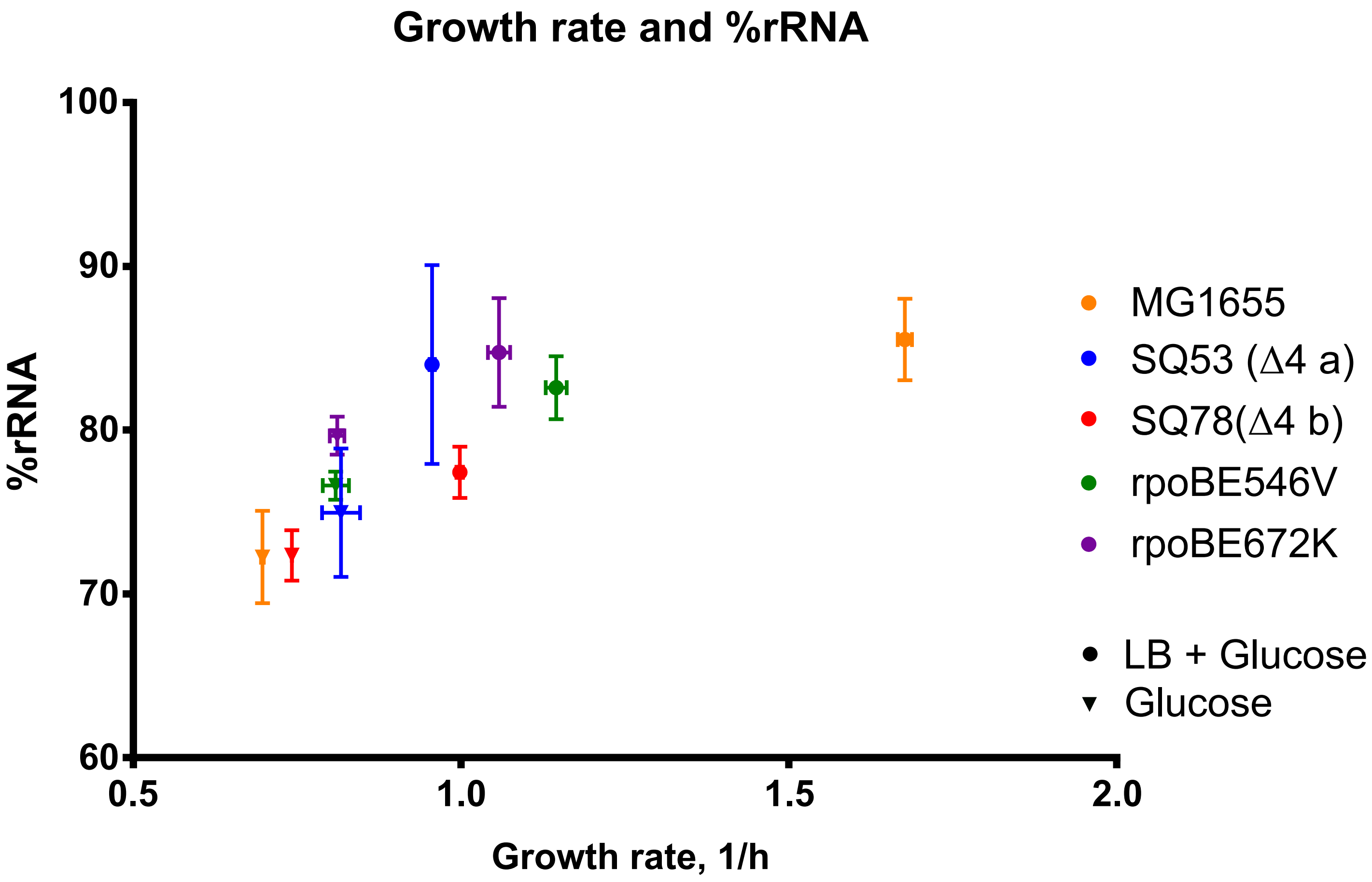

Figure S5. Related to figure 2. %rRNA and growth rates . The % of ribosomal RNA was quantified for selected mutants. rRNA content is increased in mutants in glucose minimal as well as their growth rates. In rich medium, rRNA is similar across strains but mutant growth rates are significantly lower than the WT.

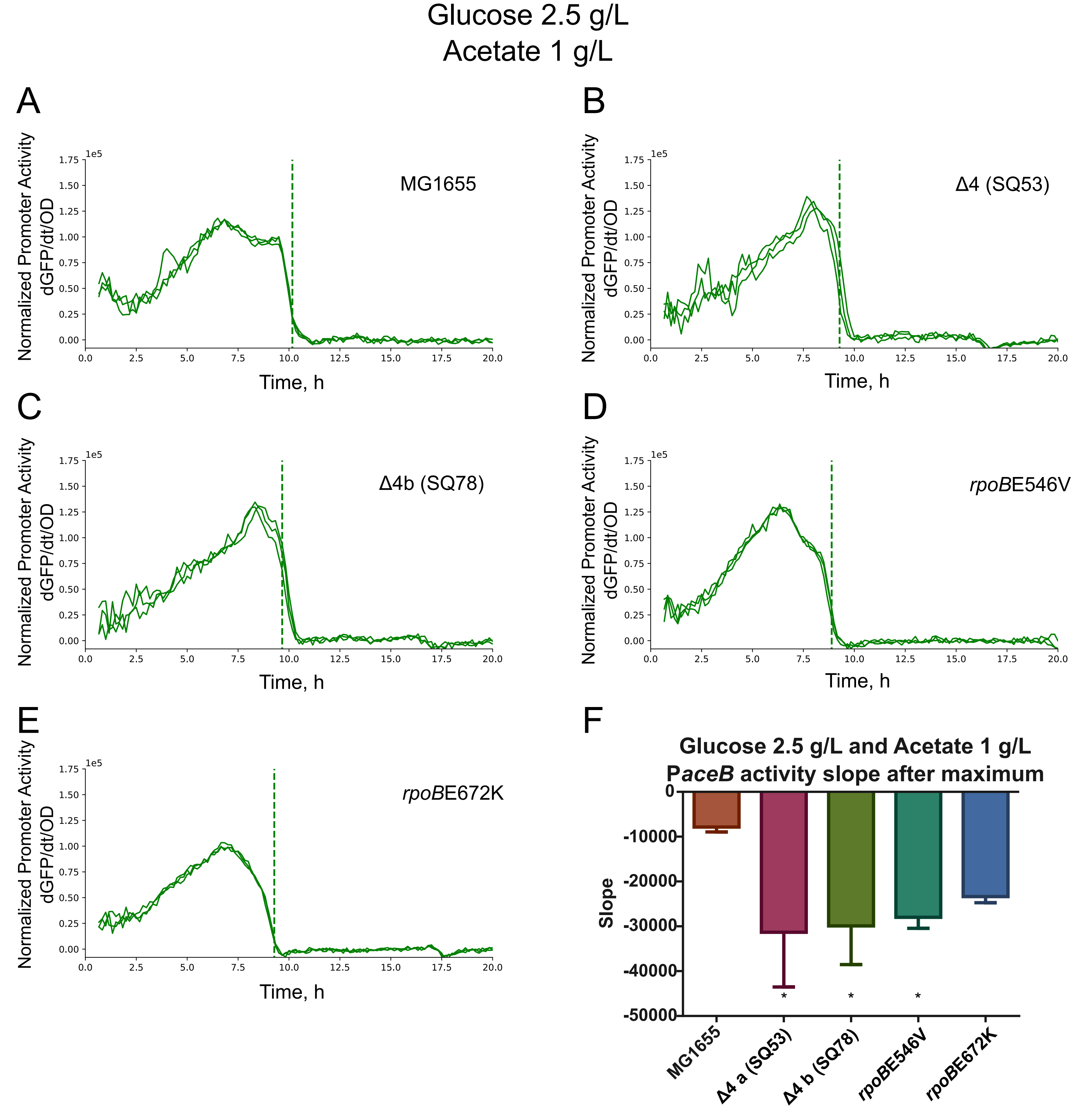

Figure S6. Related to Fig. 3. A-E, PaceB activity in glucose (2.5 g/L) and acetate (1 g/L). Dashed line represents time at growth shifts. F, PaceB activity slopes after maximum. * represents significant differences at 95% confidence.

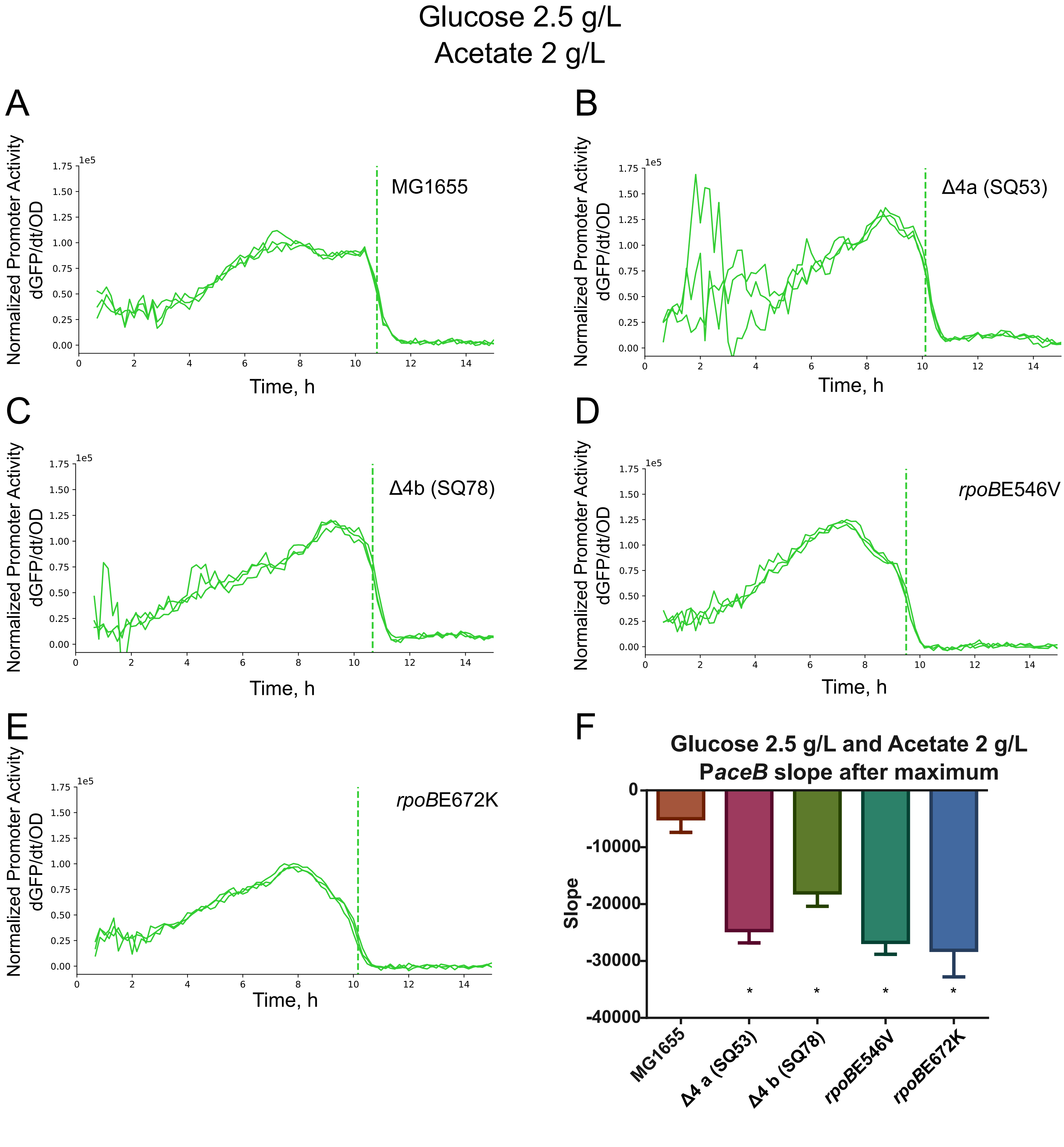

Figure S7. Related to Fig. 3. A-E, PaceB activity in glucose (2.5 g/L) and acetate (2 g/L). Dashed line represents time at growth shifts. F, PaceB activity slopes after maximum. * represents significant differences at 95% confidence.

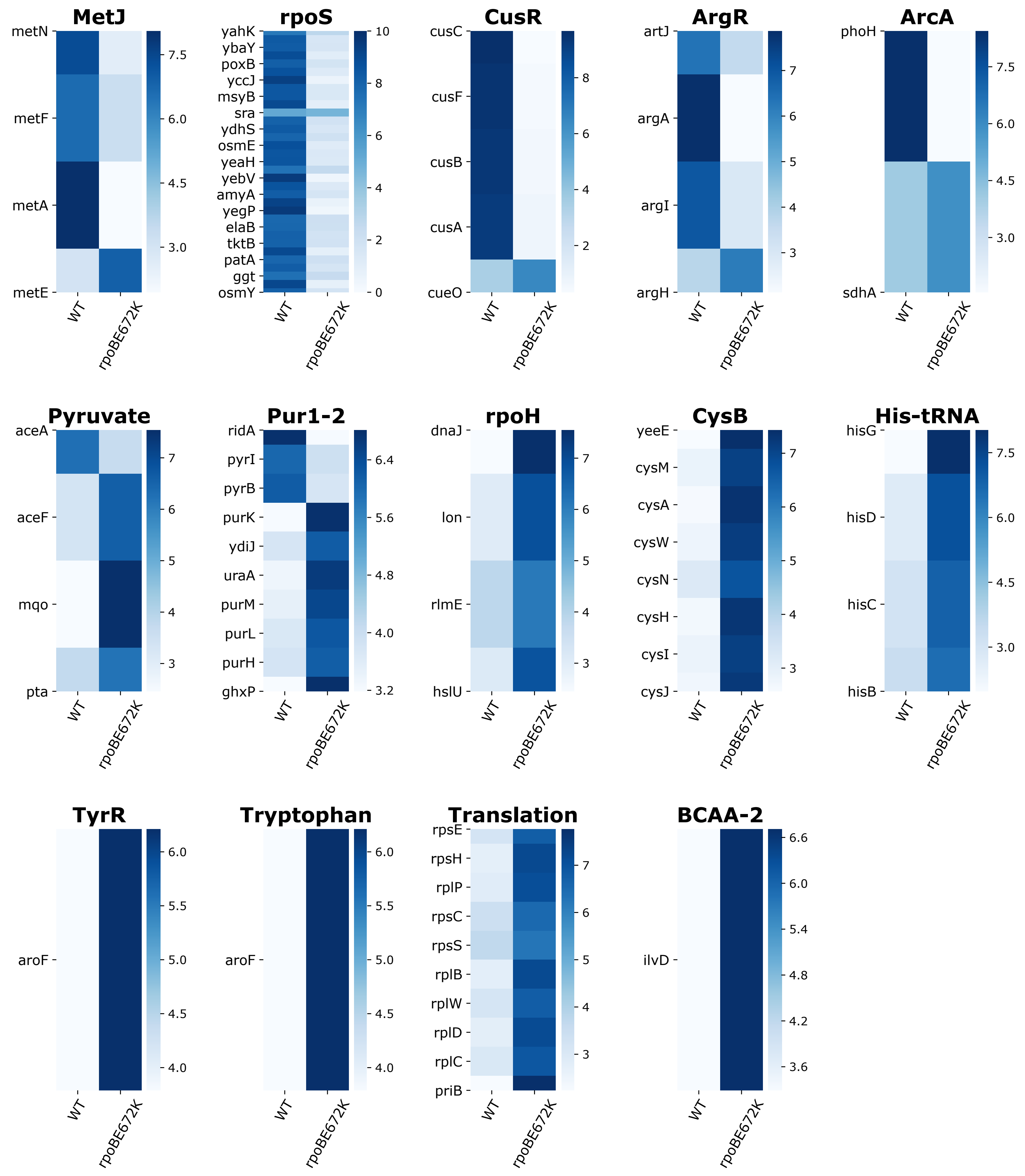

Figure S8. Related to Fig. 6A. The iModulon categorization of rpoBE672K 's DEGs. iModulons show a Fear vs. Greed response, just like SQ53. The number of DEGs in this strain allows for more iModulons to be associated with the transcriptomic data, all of which are shown here. The three iModulons for SQ53 are those presented in figure 6.
